# Electrophysiological and biochemical effect of zinc oxide nanoparticles on heart functions of male Wistar rats

**DOI:** 10.1038/s41598-024-65189-9

**Published:** 2024-07-04

**Authors:** Aida Ahmed Hussein, Eman Raafat Moatamed, Mohamed Mahmoud El-desoky, Zakaria El Khayat

**Affiliations:** 1 Zoology Department, Faculty of Science, Suez University, Suez, Egypt; 2 Physics Department, Faculty of Science, Suez University, Suez, Egypt; 3 Medical Biochemistry Laboratory, National Research Center, Cairo, Egypt

**Keywords:** ZnO NPs, ZnO bulk form, Cardiotoxicity, Electrocardiogram, Creatine kinase-MB, Lactate dehydrogenase, Oxidative stress, Antioxidant biomarkers, Zinc concentration, Heart tissues, Biochemistry, Chemical biology, Physiology, Biomarkers, Cardiology, Materials science, Nanoscience and technology

## Abstract

Zinc oxide nanoparticles (ZnO NPs) are one of the most abundantly used nanomaterials in cosmetics and topical products, and nowadays, they are explored in drug delivery and tissue engineering. Some recent data evidenced that they are responsible for cardiotoxic effects and systemic toxicity. The present study aimed to investigate the toxic effect of ZnO NPs (39 nm) on the heart of Wistar rats and to perform a dose–response relationship using three different dose levels (25, 50, 100 mg/kg bw) of ZnO NPs on the electrocardiogram (ECG) readings, the levels of biochemical function parameters of heart, and the oxidative stress and antioxidant biomarkers. Furthermore, zinc concentration level and histopathological examination of heart tissues were determined. ZnO NPs showed a dose-dependent effect, as the 100 mg/kg bw ZnO NPs treated group showed the most significant changes in ECGs parameters: R–R distance, P–R interval, R and T amplitudes, and increased levels of heart enzymes Creatine Kinase- MB (CK-MB) and Lactate dehydrogenase (LDH). On the other hand, elevated zinc concentration levels, oxidative stress biomarkers MDA and NO, and decreased GSH levels were found also in a dose-dependent manner, the results were supported by impairment in the histopathological structure of heart tissues. While the dose of 100 mg/kg bw of ZnO bulk group showed no significant effects on heart function. The present study concluded that ZnO NPs could induce cardiac dysfunctions and pathological lesions mainly in the high dose.

## Introduction

In recent decades, nanotechnology has drawn the attention of worldwide scientific researchers due to its expanding production and demand for industrial and biomedical applications^1^. Zinc oxide nanoparticles (ZnO NPs) are the most widely used metal oxide nanomaterials owing to their broad industrial and household applications, as in pigments, cosmetics, and electronic devices, due to their photocatalysts and optical properties^2,3^. ZnO NPs can readily absorb ultraviolet (UV) A and B radiations and are transparent, which makes them ideal for use in cosmetics (sunscreens, foot care, ointments, and topical products)^4^. Currently, their global production is valued at one million tons per year^5^**.**

ZnO NPs are significantly preferred over bulk ZnO particles in the food packaging industry due to their antibacterial, and antimicrobial properties, as well as their enhanced chemical reactivity^6^. Along with nanosilver (Ag NPs) and copper oxide (CuO) particles, they are used in consumer and household products to prevent the undesirable growth of bacteria, fungi, and algae^7^. There is an increasing need for novel nanoparticles in several medicinal fields such as tissue engineering, drug delivery, and drug investigating research^8^. ZnO NPs are frequently used in biomedical and pharmaceutical probes for drug delivery, as they have been recently extensively examined for their anticancer activity, moreover, they are now well-known for their cellular physiological, and neuromedical applications^9^.

However, increased production and consumption of ZnO NPs in industrial and household products has caused secondary pollution to the environment due to toxic effects on ‘nontarget’ organisms^6^. Despite the broad applications of NPs, their interactions with biological systems are relatively unclear^10^, possibly due to several experimental challenges faced while assessing the toxicity of nanomaterials^11^. Reports have shown that inhaled or ingested ZnO NPs in animal models could induce translocation into the blood circulation, resulting in distribution to vital organs such as the heart and liver^12^. The higher solubility of ZnO NPs in living systems compared to their bulk forms increases the accumulation of zinc ions at concentrations high enough to induce oxidative stress and reactive oxygen species (ROS) production^13^. In our previous work, we demonstrated increased toxicity of ZnO NPs by showing that intraperitoneal (i.p.) injection with 39 nm-sized ZnO NPs induced significant hepatic dysfunction and pathological lesions in male Wistar rats. Moreover, we observed an elevation in oxidative stress biomarkers in a dose-dependent manner, whereas no toxic effect was noticed after injecting ZnO bulk form (5 microns)^14^. We suggested that ZnO NPs induced ROS production, which disrupted intracellular metabolic activities and antioxidant systems by damaging lipids, carbohydrates, proteins, and DNA in living cells^15^. Studies showed that ZnO NPs injected via the i.p. route showed more toxic effects on the livers of mice than those injected via the gastrointestinal route, suggesting that the type of exposure route determines the toxicological effects of the ZnO NPs^16^.

Cardiotoxicity is described as the disruption of the normal function of the heart muscles and tissues, including disturbances in the heart’s electrophysiology, resulting in its inability to pump a sufficient amount of blood throughout the body^17^. The first definition of myocardial infarction, proposed by the World Health Organization (WHO), was associated with increased levels of total creatine kinase (CK), CK‑myocardial band (CK-MB), and lactate dehydrogenase (LDH) in the blood^18^**.** Moreover, in 2005, the United States Food and Drug Administration authorized the use of a human electrocardiogram (ECG) to evaluate the cardiotoxicity of new drugs^19^.

Recent studies suggested that metal oxide nanoparticles used in agricultural pesticides cause heavy metal accumulation, resulting in cardiotoxicity and oxidative stress in humans and animals^20^. Inhalation of ZnO NPs induces cardiopulmonary injury in occupational workers handling these nanomaterials^21^. Additionally, injection with high doses of ZnO NPs (600 mg/kg and 1 g/kg body weight) for 5 days increased serum cardiac injury markers CK-MB, myoglobin, and troponin, which indicated its cardiotoxic side effects such as myocardial infarction^22^. Furthermore, ZnO NPs were reported to reduce cell survival and impact the metabolism of cardiomyocytes in mice and neonatal rats^23^.

To date, the Organization for Economic Co-operation and Development reported that in addition to the standard tests, additional testing is required for ZnO nanomaterials based on size, PH, particle dissolution, and differentiation between nano and bulk sizes to ensure their safety toward humans and the environment^24^**.** Although studies have evaluated the toxic effects of ZnO NPs on heart tissues, however their effects on the biochemistry and electrophysiology of the heart have not been well-researched in vivo. Rats and mice are the most commonly used experimental animals for cardiotoxicity evaluation and electrophysiological studies^25^. This study aimed to assess the in vivo toxicity of ZnO NPs (39 nm) on electrophysiological and biochemical heart functions of male Wistar rats, with also a comparison between the effects of ZnO NPs and their bulk/micron forms (particle size = 5 μm).

## Material and methods

### Preparation and characterization of ZnO NPs

The ZnO NPs (nanorods) powder was prepared in the Department of Physics, Faculty of Science, Suez University, Suez, Egypt, using microwave irradiation. The reagents and the ZnO bulk material (particle size = 5 μm) were purchased from Sigma-Aldrich, Germany. The ZnO NPs and bulk material suspensions were freshly prepared using physiological saline (0.9% sodium chloride). The details about the preparation and characterization of the ZnO nanomaterials and bulk suspensions are available in our previous study^14^.

## Animals and experimental design

### Experimental groups

We purchased 30 healthy adult male Wistar rats (8–10 weeks old, 180–200 gm each) from the Egyptian National Research Center (NRC). They were housed in suitable polypropylene cages under standard laboratory conditions for at least one week before the experiment for acclimatization. These animals received an adequate staple diet of 60% ground corn and 40% ground beans. Food and water were provided ad libitum. The body weight and behavior of the rats were carefully recorded to observe the possible appearance of clinical signs following the treatment. The rats were divided into five equal groups (n = 6/group), and each group received a different treatment. The rats in each group were given i.p. injections every other day for ten days with the following: *Control group*: 0.5 ml of 0.9% normal saline as a vehicle; (2) *Bulk group*: ZnO bulk suspensions at a dose of 100 mg/kg bw; T1, T2, and T3 (ZnO NP-treated) groups: ZnO NPs suspension at doses of 25, 50 and 100 mg/kg bw, respectively.

### Ethical approval

All of the animals used in the experiments received adequate care and the study protocol performed on animals complies with the guidelines of the National Institutes of Health and Ethical Conduct for Use and Care of Animals in Research Ethics Committee REC in Faculty of Medicine, Suez University (approval number: 19323). All methods were performed in accordance with the relevant guidelines (https://arriveguidelines.org).

### Electrophysiological study

#### Anesthesia

Rats’ electrocardiogram recording was done in the five treated groups (one day before blood sampling). All treated rats were anesthetized with a dose of 1.25 g/kg bw urethane (Sigma-Aldrich, Germany), using i.p. injections, which is a widely used surgical anesthetic, since urethane can provide deep anesthetic effects while maintaining normal cardiovascular reflexes in the treated animals^28^**.**

#### ECG recordings

The ECG was recorded in the rats using a three-channel Digital ECG (ECG-300G, Biocare Bio-Medical co, Ltd, Germany) with a chart speed of 50 mm/second. The rats were placed in a dorsal position on a wooden platter, and four electrodes (Lead II) were attached to the skin of the forelimbs and hindlimbs^29,30^. Even though we used needle electrodes for the ECG recordings, the process was conducted in a quiet room to reduce stress. Gentle restrain and special care were used to obtain reliable readings from the rats.

#### Recorded ECG parameters

To assess the myocardial activity, we measured the heart rate (HR) and different ECG parameters such as conduction time (P–R interval), depolarization, and repolarization voltage (R and T waves)^31^. The HR was measured and calculated from the R–R interval. The changes were represented as chronotropic effects. The P–R interval (conduction time) was measured from the beginning of the P-wave to the start of the QRS complex, and the changes in the conduction times are called dromotropic effects. The amplitude of both R and T waves was measured from the isoelectric line up to the highest point of the waves^32^**.**

### Sample collection and tissue preparation

After the end of the experiment, the rats were fasted overnight and weighed. Then, under the previous injection of urethane anesthesia blood samples were drawn from the retro-orbital venous plexus of the eyes using a capillary tube according to a previously described method^26^. Some of the blood samples were allowed to sit for 1 h at room temperature and then centrifuged at 2000×*g* for 3 min to collect the serum, which was then stored at − 22 °C for further biochemical assays. Finally, the animals were euthanized, and their hearts were excised and divided into two halves. The first half was fixed instantly in 10% neutral-buffered formalin for 2 days and used for histopathological examinations, whereas the other half was frozen for bioassays (− 80 °C). The frozen tissues were cut into small pieces and homogenized using 10 ml of 100 mM cold potassium phosphate buffer with 1 mM EDTA (pH 7.4) as previously described^27^. Then, the homogenate was centrifuged at 12,000×*g* for 30 min at 4 ℃, and the supernatant was removed to measure oxidative stress, antioxidant parameters, and zinc content.

### Biochemical studies

#### Heart function biomarkers

Cardiac enzymes such as creatine kinase-MB (CK-MB) and lactate dehydrogenase (LDH) were determined by enzymatic colorimetric method using commercial kits (SPINREACT Co., S.A.U, Spain).

#### Heart oxidative stress and antioxidant parameters

##### Determination of tissue-reduced glutathione (GSH)

GSH was measured in the heart tissue of rats using a commercially available kit (Biodiagnostic, Egypt) as described previously^33^.

##### Determination of tissue nitric oxide (NO)

NO was determined in the heart tissues of rats using a colorimetric kit (Bio diagnostic, Egypt) as described previously^34^.

##### Determination of tissue malondialdehyde (MDA)

MDA was determined in the heart tissues of rats calorimetrically using a commercial kit (Biodiagnostic, Egypt) according to a previous method^35^.

#### Zinc ion concentration in heart tissues

The concentration of zinc ions (Zn^++^) in each heart tissue of rats was measured using **a** Perkin Elmer- A Analyst 100 spectrophotometer using a previously discussed method^36^**.**

### Histomorphological examination

Paraffin-embedded heart samples (5 μm sections) were stained with hematoxylin and eosin (H&E) and observed under a light microscope (CX21Olympus^®^, Japan) at a magnification of 400× to observe the histomorphology of the liver and heart. All slides were captured at a resolution of 10 MP (megapixels) by a UIS optical system (Universal Infinity System, Olympus^®^)^37^.

### Statistical analysis

All data were coded, tabulated, and statistically analyzed using IBM SPSS statistics (Statistical Package for Social Sciences) software version 21 (IBM Corp., Chicago, USA). First, the normal distribution of the data was analyzed using the Shapiro–Wilk test to determine whether the results should be analyzed parametrically or non-parametically. Furthermore, the homogeneity of variances was checked using Levene’s tests. The results were expressed as mean ± standard error. Comparison between groups was performed using a one-way analysis of variance if the data were normally distributed. The post hoc Duncan and Games–Howell tests were then used for homogeneous and nonhomogeneous variances, respectively. Additionally, for nonnormal distributed data, we performed the Kruskal–Wallis test followed by the Mann–Whitney *U*-test. P-value < 0.05 was considered significant, whereas p-values < 0.01 or 0.001 were considered highly significant.

### Ethics declarations

In accordance with ethical standards and guidelines for the use of animals in research, the animal experiments conducted in this study were approved by Research Ethics Committee REC in Faculty of Medicine, Suez University (approval number: 19323). All procedures involving animals were performed in compliance with relevant laws and regulations, and every effort was made to minimize any potential discomfort or distress to the animals. Prior to the commencement of the study, informed consent was obtained from the respective authorities, and proper measures were taken to ensure the humane and ethical treatment of the animals throughout the duration of the experiments. The details of the ethical approval and procedures are outlined in the "Methods" section of this manuscript, as required by the journal’s submission guidelines (https:// arrive guidelines. org).

### Consent to participate

All authors agree to participate in this study**.**

## Results

### Experimental electrophysiology of control and treated groups

**Figure 1:**
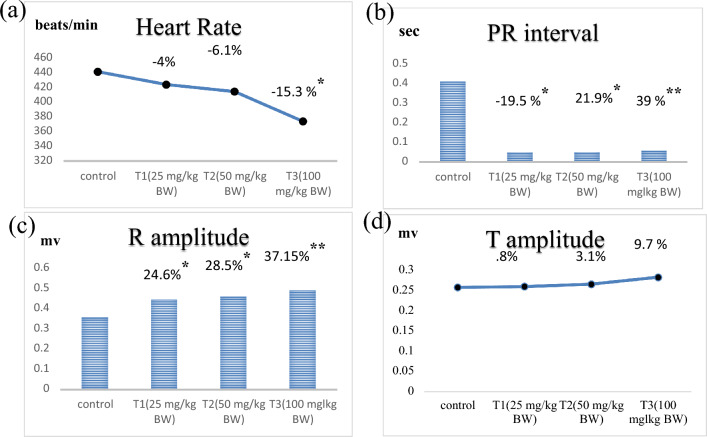
Represents the dose–response relationship in Electrocardiogram parameters following the treatment of ZnO NPs at three different doses. ZnO NPs: zinc oxide nanoparticles; ZnO NPs three doses: 25 mg/kgbw, 50 mg/kgbw, and 100 mg/kgbw. (**a**) HR: heart rate. (**b**) PR interval: conduction time. (**c**) R amplitude: depolarization voltage. (**d**) T amplitude: repolarization voltage. % Percentage of change as compared to the control group. *p < 0.05, **p < 0.001, ***p < 0.0001 compared to control group.

The ECG readings of rats (Table 1) showed that the T3 group (100 mg/kg BW) showed a significant decrease in HR (by 15.3%), whereas the T2 and T1 rats (50 and 25 mg/kg BW) showed nonsignificant decreases in HR (by 6.1% and 4%, respectively) compared to the control group. These changes in HR indicated a dose-dependent increase in ZnO NP toxicity (Fig. 1a). Conversely, the bulk group rats showed a nonsignificant decrease in HR (by 1.5%) compared to the control. Comparison between ZnO NPs and the bulk groups showed that the T3 group showed a significant decrease in HR (− 13.9%), whereas T2 and T1 showed nonsignificant decreases (− 4.6% and − 2.4%, respectively).

**Table 1:** Electrocardiogram (ECG) parameters of control, bulk, and the three ZnO NP-treated groups (T1, T2, and T3).

			Animal groups				
			Control	Bulk	T1	T2	T3
HR (beats/min)	Mean ± SE		441 ± 6.4	434 ± 8.0	423.5 ± 2.2	414 ± 7	373.6 ± 4.2^a^*
	% of change	A		− 1.5%	− 4%	− 6.1%	− 15.3%
		B			− 2.4%	− 4.6%	− 13.9%
PR interval (s)	Mean ± SE		0.041 ± 0.004	0.048 ± 0.004^a^*	0.049 ± 0.003^a^*	0.05 ± 0.005^a^*	0.057 ± 0.001^ab^**
	% of change	A		+ 17%	+ 19.5%	+ 21.95%	+ 39%
		B			+ 2.08%	+ 4.16%	+ 18.75%
R-amplitude (mv)	Mean ± SE		0.358 ± 0.02	0.436 ± 0.02 ^a^*	446 ± 0.02^a^*	0.46 ± 0.03^a^*	0.491.02^a^**
	% of change	A		+ 21.7%	+ 24.6%	+ 28.5%	+ 37.15%
		B			+ 2.14%	+ 3.13%	+ 10%
T-amplitude (mv)	Mean ± SE		0.258 ± 0.02	0.259 ± 0.03	0.26 ± 0.02	0.266 ± 0.02	0.283 ± 0.016
	% of change	A		+ 0.4%	+ 0.8%	+ 3.1%	+ 9.7%
		B			+ .38%	+ 2.7%	+ 9.2%

A: percentage of change compared to the control group.

B: percentage of change compared to the bulk group.

*HR* heart rate, *PR interval* conduction time, *R-amplitude* depolarization voltage, *T-amplitude* repolarization voltage, *ZnO NPs* zinc oxide nanoparticles, *ZnO NP-treated groups* T1: 25 mg/kg BW, T2: 50 mg/kg BW, T3: 100 mg/kg BW, *Bulk* ZnO bulk treated group (100 mg/kg BW), *SE* standard error.

*p < 0.05, **p < 0.001, ***p < 0.0001 compared to the control group.

Values are shown as mean ± SE (n = 6), and the superscripts in different letters represent the following: ^a^significant compared to the respective control group, ^b^significant compared to the bulk group.

Furthermore, the ECG results confirmed that the application of ZnO NPs induced a highly significant increase in the P-R intervals in the T3 group (by 39%), whereas the increases in the T2 and T1 groups were significant (by 21.95% and 19.5%, respectively) compared to the control group. This reveals the dose-dependent effect of the ZnO NPs (Fig. 1b). The ECGs of the rats in the bulk group displayed a significantly higher P-R interval (by 17%) than in the control group. Moreover, when we compared the effects in the ZnO-NP-treated and bulk groups, we observed a significantly higher P-R interval in the T3 group (by 18.75%), whereas the increases in the T2 and T1 groups were nonsignificant (by 4.16% and 2.08%, respectively).

The ECG data also showed that treatment with ZnO NPs caused a dose-dependent increase in the R-amplitude of rats’ ECGs in the T1, T2, and T3 groups (Fig. 1c). It was significantly higher in all the groups (37.15%, 28.5% and 24.6% in T3, T2, and T1, respectively) compared to the control group. Furthermore, the bulk group showed a significant increase in R amplitude (21.78%) compared to the control. Comparison between the ZnO NP-treated and bulk groups revealed a nonsignificant increase in R amplitude in the T3, T2, and T1 (by 10%, 3.13%, and 2.14%, respectively).

The results also indicated a dose-dependent yet nonsignificant increase in T-amplitude in the ZnO NP-treated groups (by 9.7%, 3.1%, and 0.8% in the T3, T2, and T1 groups, respectively) compared to the control group (Fig. 1d). Moreover, the bulk group showed a nonsignificant increase by 0.4% compared to the control group. Compared to the bulk group, the ZnO NP-treated groups showed nonsignificant increases in T-amplitude (by 9.2%, 2.7%, and 0.38% in the T3, T2, and T1 groups, respectively).

**Plate 1:**
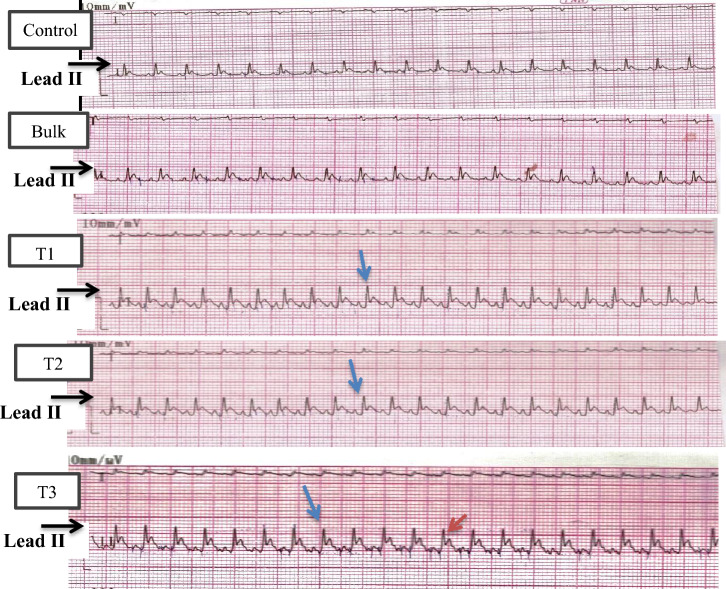
ECGs recording charts from control, bulk, T1, T2, and T3 treated group (results are demonstrated in lead II). ZnO NPs treated groups: T1: 25 mg/kgbw; T2: 50 mg/kgbw; T3: 100 mg/kgbw; bulk: ZnO bulk treated group (100 mg/kgbw). Blue rightward arrow: increase in depolarization voltage (R-amplitude). Red leftward arrow: increase in repolarization voltage (T-amplitude).

**Plate 2:**
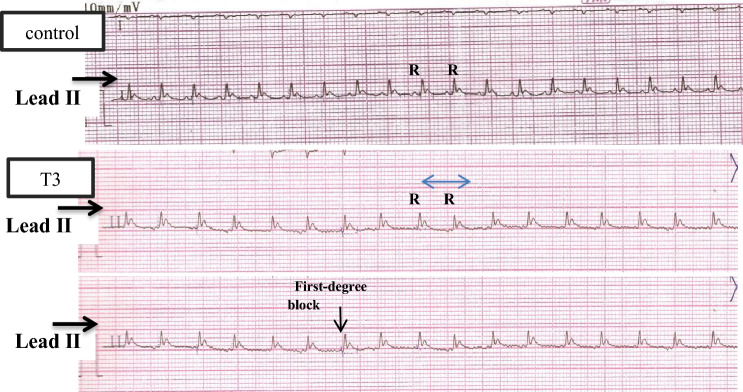
Examples of cardiac disorders from ECG recording charts of T3-treated rats compared to control ECG charts, the cardiac disorders represented by first-degree block and Bradycardia. T3: ZnO NPs (100 mg/kgbw) treated group. Black downward arrow: first-degree block (absence of P-wave). Blue leftward rightward arrow: R–R interval showing (bradycardia).

From the changes observed in the ECG parameters between control and ZnO NP-treated groups, such as R and T amplitudes (Plate 1), we deduced that the rats in the T3 group exhibited cardiac disorders as represented by the increased R-R distance (bradycardia), longer P-R intervals and missing P waves (first-degree block) (Plate 2).

### Heart function enzymes

**Figure 2:**
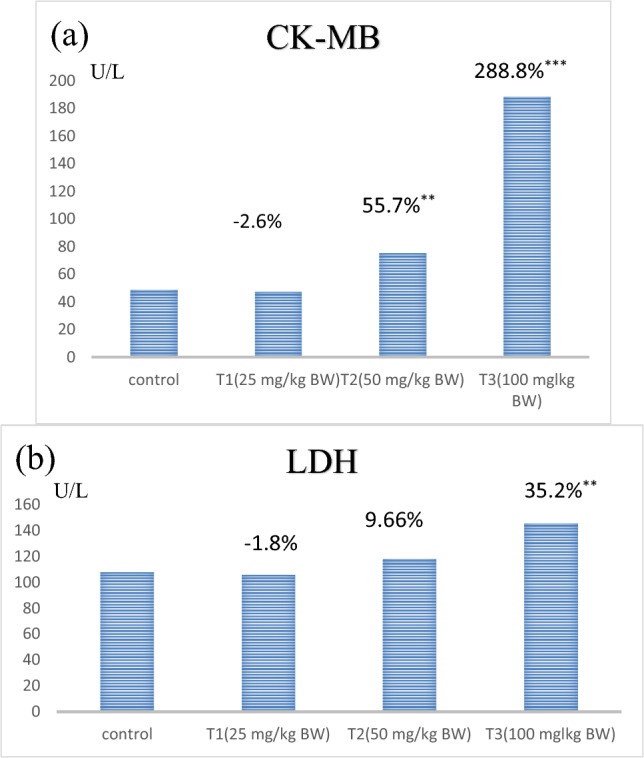
Represents the dose–response relationship in heart function parameters following the treatment of ZnO NPs at three different doses. ZnO NPs: zinc oxide nanoparticles; ZnO NPs three doses: 25 mg/kgbw, 50 mg/kgbw, and 100 mg/kgbw. (**a**) CK-MB: creatine kinase-MB. (**b**) LDH: lactate dehydrogenase. % Percentage of change as compared to the control group. **p < 0.001, ***p < 0.0001 compared to control group.

The biochemical data on heart function biomarkers (Table 2) demonstrated that the serum levels of CK-MB in the ZnO NP-treated rats showed an extremely significant increase (by 288.8%) in the T3 group, whereas that in the T2 group showed a highly significant increase (by 55.7%). Conversely, in the T1 group, there was a nonsignificant decrease (by − 2.68%) compared to the control group. This depicted the dose-dependent response in CK-MB levels in the three ZnO NP-treated groups (Fig. 2a). The data also revealed that ZnO bulk-treated rats produced a nonsignificant decrease in CK-MB levels by − 8% compared to the control group. Compared to the bulk group, the data showed a highly significant increase in CK-MB in both T3 and T2 groups by 322.7% and 69.3%, respectively, whereas the T1 rats demonstrated a nonsignificant increase of 5.8%.

**Table 2:** Levels of heart enzymes, CK-MB, and LDH in control, bulk, and three ZnO NP-treated groups.

			Animal groups				
			Control	Bulk	T1	T2	T3
CK-MB (U/L)	Mean ± SE		48.38 ± 0.56	44.5 ± 1.08	47.08 ± 0.73	75.33 ± 1.17^ab^**	188.1 ± 0.87^ab^***
		A		− 8%	− 2.68%	+ 55.7%	+ 288.8%
		B			+ 5.8%	+ 69.3%	+ 322.7%
LDH (U/L)	Mean ± SE		107.6 ± 1.6	108.9 ± 0.52	105.6 ± 0.66	118 ± 1.12	145.5 ± 1.6^ab^**
		A		+ 1.2%	− 1.8%	+ 9.66%	+ 35.2%
		B			− 2.97%	+ 8.35%	+ 33.6%

*CK-MB* creatine kinase-MB, *LDH* lactate dehydrogenase, *ZnO NP-treated groups* T1: 25 mg/kg BW, T2: 50 mg/kg BW, T3: 100 mg/kg BW, *bulk* ZnO bulk treated group (100 mg/kg BW), *SE* standard error.

*p < 0.05, **p < 0.001, ***p < 0.0001 compared to control group.

Values are shown as mean ± SE (n = 6) and the superscript in different letters indicates the following: ^a^significant compared to the respective control group. ^b^Significant compared to the bulk group. A: percentage of change as compared to the control group. B: percentage of change as compared to the bulk group.

The data also showed that serum LDH levels were significantly increased in the T3 group by 35.2%. However, the increase in the T2 group was nonsignificant (by 9.66%), whereas the T1 group showed a nonsignificant decrease (by − 1.8%) compared to the control. This clarifies the dose-dependent effect of ZnO NPs (Fig. 2b). While bulk ZnO-treated rats produced a nonsignificant increase (by 1.2%) compared to the control. Compared to the bulk group, the LDH levels were significantly higher in the T3 group (33.6%). Furthermore, the T2 group showed a nonsignificant increase in LDH (8.35%), whereas T1 showed a nonsignificant decrease (− 2.97%).

### Oxidative stress and antioxidant biomarkers levels in the heart tissues

**Figure 3:**
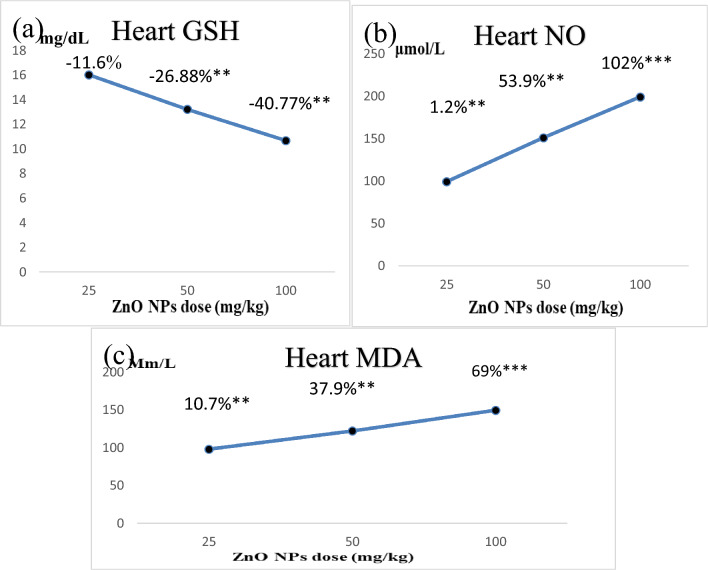
Represents the dose–response relationship in heart oxidative stress and antioxidant parameters following the treatment of ZnO NPs at three different doses. ZnO NPs: zinc oxide nanoparticles; ZnO NPs three doses: 25 mg/kgbw, 50 mg/kgbw, and 100 mg/kgbw; (**a**) GSH: heart reduced glutathione, (**b**) NO: heart nitric oxide, and (**c**) MDA: heart malondialdehyde. % Percentage of change as compared to the control group. **p < 0.001,***p < 0.0001 compared to control group.

The data on the oxidative stress and antioxidant biomarkers levels demonstrated that ZnO NP-treated rats showed a significant decrease in reduced glutathione (GSH) levels in rats in T3 and T2 (− 40.77% and − 26.88%, respectively) compared to the control group (Table 3). Conversely, the rats in the T1 group showed a nonsignificant decrease in GSH levels (− 11.6%). This indicated the dose-dependent effect of ZnO NPs (Fig. 3a). However, the bulk group showed a nonsignificant increase in the GSH levels (2%) compared to the control. When we compared the GSH levels in the ZnO NP-treated and the bulk groups, the GSH levels in the T3 and T2 groups were significantly lower (by − 42.16% and − 28.6%, respectively), whereas T1 showed a nonsignificant decrease (− 13.5%).

**Table 3:** Changes in the oxidative stress and antioxidant markers, including GSH, NO, and MDA levels, in the control and treated groups.

			Animal groups				
			Control	Bulk	T1	T2	T3
H. GSH (mg/dL)	Mean ± SE		18.15 ± 0.61	18.5 ± 0.4	16.0 ± 0.73	13.2 ± 0.6^ab^**	10.66 ± 0.62^ab^**
	% of change	A		+ 2.0%	− 11.6%	− 26.88%	− 40.77%
		B			− 13.5%	− 28.6%	− 42.16%
H. NO (μmol/L)	Mean ± SE		98.0 ± 2.0	87.66 ± 2.3	99.16 ± 1.35	150.9 ± 2.9^ab^**	198.8 ± 2.7^ab^***
	% of change	A		− 10.5%	+ 1.2%	+ 53.9%	+ 102%
		B			+ 11.5%	+ 63.2%	+ 111.2%
H. MDA (Μm/L)	Mean ± SE		88.5 ± 0.99	86.5 ± 1.47	98 ± 0.73	122.1 ± 1.4^ab^**	149.6 ± 1.17^ab^***
	% of change	A		− 2.25%	+ 10.7%	+ 37.9%	+ 69%
		B			+ 12.5%	+ 38.9%	+ 68.8%

*GSH* reduced glutathione, *NO* nitric oxide, *MDA* malondialdehyde, *ZnO NP-treated groups* T1: 25 mg/kg BW, T2: 50 mg/kg BW, T3: 100 mg/kg BW, *bulk* ZnO bulk treated group (100 mg/kg BW), *SE* standard error.

*p < 0.05, **p < 0.001, ***p < 0.0001 compared to control group.

Values are shown as means ± SE (n = 6) and the superscripts in different letters indicate the following**:** ^a^significant compared to the respective control group. ^b^Significant compared to the bulk group. A: percentage of change as compared to the control group. B: percentage of change as compared to the bulk group.

The data also confirmed the dose-dependent effect of ZnO NPs on the nitric oxide (NO) levels in the heart tissues of the treated rats (Fig. 3b). The NO levels showed a highly significant increase in the T3 group (102%) and a significant increase in the T2 group (53.9%). Whereas the T1 group showed a nonsignificant increase (1.2%) compared to the control. However, the NO levels in heart tissues of the bulk group showed a nonsignificant decrease (− 10.5%) compared to the control. Comparison of the NO levels in the ZnO NP-treated and the bulk groups revealed a highly significant increase in the T3 and T2 groups (111.2% and 63.2%, respectively), whereas the T1 group showed a nonsignificant increase (11.5%) compared to the bulk group.

We observed that the malondialdehyde (MDA) levels in the heart tissues of the ZnO NP-treated rats were highly significantly increased in the T3 group (69%), significantly increased in T2 (37.9%), and nonsignificantly increased in T1 groups (10.7%), proving the dose-dependent effect of ZnO NPs (Fig. 3c) However, the bulk group showed a nonsignificant decrease in MDA levels (− 2.25%) compared to the control group. Furthermore, compared to the bulk group, the T3, T2, and T1 groups showed highly significant, significant, and nonsignificant increases in MDA levels (by 68.85%. 38.9%, and 12.5%, respectively).

### Zinc ion concentration in the heart

**Figure 4:**
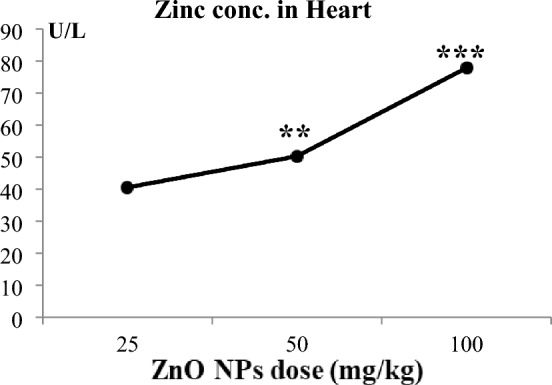
Represents the dose–response relationship in heart zinc ions concentration following the treatment of ZnO NPs at three different doses. ZnO NPs: zinc oxide nanoparticles; ZnO NPs three doses: 25 mg/kgbw, 50 mg/kgbw, and 100 mg/kgbw; *conc.* concentration. % Percentage of change as compared to the control group. **p < 0.001,***p < 0.0001 compared to control group.

We assessed the zinc ions (Zn^++^) concentrations in the heart tissues of rats (Table 4). The increases in the Zn^++^ content in the T3, T2, and T1 groups were highly significant (103.6%), significant (31.55%), and nonsignificant (6.1%), respectively. Again, this indicated the dose-dependent effect of ZnO NPs on the zinc ions concentrations (Fig. 4). The bulk group showed a nonsignificant increase in Zn^++^ content by 4.55% compared to the control group. Compared to the bulk group, the increases in Zn concentration were nonsignificant in T1 (1.5%), significant in the T2 group (25.8%), and highly significant in T3 (94.7%).

**Table 4:** Zinc ion concentrations in heart tissues in control and treated groups.

			Animal groups				
			Control	Bulk	T1	T2	T3
Zn^++^ conc. in heart (U/L)	Mean ± SE		38.16 ± 1.0	39.9 ± 0.61	40.5 ± 0.67	50.2 ± 1.19^ab^**	77.7 ± 1.83^ab^***
	A			+ 4.55%	+ 6.1%	+ 31.55%	+ 103.6%
	B				+ 1.5%	+ 25.81%	+ 94.7%

*Zn*^*++*^ zinc ions, *conc.* Concentration, *ZnO NP-treated groups*T1: 25 mg/kg BW, T2: 50 mg/kg BW, T3: 100 mg/kg BW, *bulk* ZnO bulk treated group (100 mg/kg bw), *SE* standard error.

*p < 0.05, **p < 0.001, ***p < 0.0001 compared to the control group.

Values are shown as means ± SE (n = 6) and the superscripts in different letters indicate the following: ^a^significant compared to the respective control group. ^b^Significant compared to the bulk group. A: percentage of change as compared to control group. B: percentage of change as compared to the bulk group.

### Histopathological observation of the heart

**Figure 5:**
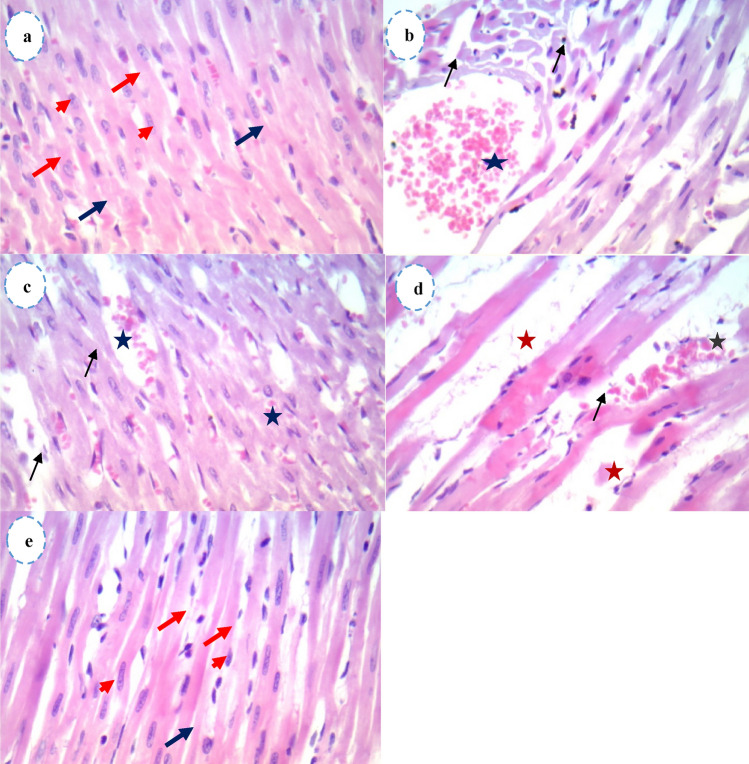
Effect of ZnO NPs and bulk form on histopathology of heart tissues of treated rats. Heart sections stained with H&E (×400), Scale bar represent 25μm (**a**) Heart tissues of control rats showing the normal appearance of cardiac myofibers, single oval and centrally located nuclei of cardiomyocytes (arrowheads), regularly arranged cardiac myofibres (red arrows), and intercalated disks (blue arrows), (**b**) heart tissues of ZnO NPs T1 (25 mg/kgbw) treated rats showing vascular congestion (black asterisk) nearby muscles looked darker and degenerated (black arrows), (**c**) heart tissues of ZnO NPs T2(50 mg/kgbw) treated rats showed marked vascular congestion (black asterisks), moreover, thickening of vessel walls. The nearby muscles looked darker and degenerated (black arrows), and mild myocyte degeneration was found (black arrows), (**d**) heart tissues of ZnO NPs T3 (100 mg/kgbw) treated rats showed marked vascular congestion (black asterisks) nearby muscles looked darker and degenerated (black arrows), and also dislocation of myofibres (red asterisks), (**e**) heart tissues of ZnO bulk (100 mg/kgbw) treated rats showed the normal structure of cardiomyocytes. Oval and centrally located nuclei of cardiomyocytes (arrowheads), regularly arranged cardiac myofibres (red arrows), and intercalated disks (blue arrows). *ZnO NPs* zinc oxide nanoparticles, *H&E* hematoxylin and eosin.

The histopathological observations in heart sections of the control rats showed normal (arrowheads) and regularly arranged cardiac myofibres (red arrows). Intercalated disks (blue arrows) were also seen (Fig. 5a). However, the cardiac sections of the T1 group showed vascular congestion (asterisks) and the nearby muscles were darker and degenerated (black arrows) (Fig. 5b). The heart sections of the T2 rats showed marked vascular congestion (asterisks), thickening of vessel walls along with darkening and degeneration (black arrows) of the nearby muscles. We also observed mild myocyte degeneration (black arrows) (Fig. 5c). Similarly, the heart sections of the T3 group showed marked vascular congestion (asterisks), and the nearby muscles were darker and degenerated (black arrows). Interestingly, we found myofibre dislocation in the cardiac muscle (red asterisks) in this group (Fig. 5d). Whereas the heart sections of the bulk group showed normal cardiomyocytes (arrowheads), regularly arranged cardiac myofibres (red arrows), and intercalated disks (blue arrows) (Fig. 5e).

## Discussion

One of the most important safety considerations in preclinical and clinical investigations is evaluating the cardiotoxicity of novel chemicals and drugs^38^. Physiologically, it has been established that increased plasma levels of CK are linked to skeletal or cardiac muscle damage, whereas increased serum LDH levels indicate myocardial infarction^22,39^. CK-MB and LDH parameters are accepted for the clinical diagnosis of myocardial infarction by the WHO^40^**.**

The biochemical data on the CK-MB and LDH levels in our study suggested that treatment with ZnO NPs increased the levels of both enzymes in a dose-dependent manner. The most significant change indicated by the highest percentage increase was implicated with the highest dose (100 mg/kg BW). An earlier study reported that oral injections with ZnO NPs at doses of 100 and 400 mg/kg BW for 28 days caused a significant increase in the serum cardiac markers levels, as well as alteration in the cardiac tissues associated with elevation in oxidative stress biomarkers^41^. Additionally, the accumulation of nanoparticles in the heart tissues was associated with increased CK and LDH levels in the blood and has been attributed to the disruption in the plasma membrane structure and increased permeability^42^**.**

Since electrical activity is a distinctive characteristic of the heart, any interruption in this activity or the contractile properties reflects pathogenesis^43^**.** ECG recordings provide a standardized method to evaluate the pharmacological and toxicological effects of drugs and chemicals on the heart^44^.

In this study, the ECG recordings of the anesthetized rats demonstrated the toxic effects of ZnO NPs on the myocardium as shown by the negative chronotropic effects (bradycardia) represented by a significant decrease in HR induced due to ZnO NP injection. We also observed a negative dromotropic effect represented by the significant prolongation in P-R, which reflects the delay in conduction velocity and disturbance in the electrical conductivity in the atria and the AV node. These effects occurred in a dose–response manner, and the most pronounced effects were seen at the highest dose (100 mg/kg bw).

We also proved that the application of ZnO NPs significantly increased the ventricular depolarization voltage demonstrated by the increased R-wave amplitude. The dose-dependent nature of this positive inotropic effect was demonstrated by the increase in contractile force of the ventricle with an increase in dosage. The dose-dependent increase in the ventricular repolarization voltage (T voltage) was also seen in the three ZnO NP-treated groups (T3, T2, and T1) although it was statistically nonsignificant compared to the control group.

Treatment with bulk ZnO did not affect the HR and T voltages in the rats as they were similar to the control rats. Although a significant increase in the P-R interval and R voltage was observed in this group, the positive inotropic effect and the ventricular contractile force were less than that seen in the ZnO NP-treated groups, which confirmed that the bulk form is safer than the NPs in terms of cardiotoxicity.

The present electrophysiological manifestations of ZnONPs, mainly at the highest dose, demonstrated an important point that the negative chronotropic and dromotropic effects are accompanied by positive inotropic effects. Moreover, they showed the presence of cardiac disorders, the most common being bradycardia and 1st-degree atrioventricular block.

Our data was consistent with a study performed on zebrafish embryos which showed significant bradycardia after being exposed to silica nanoparticles. These effects developed with the increase in nanomaterial concentration^45^**.** Moreover, ZnO NPs were reported to decrease HR and cell contractility in human cardiomyocytes^4^**.**

The AV node and His-bundle typically form the only electrically active connection between atria and ventricles. Therefore, the presence of bradycardia and P-R interval elongation indicates a possible lesion in the sinoatrial (SA), atrioventricular (AV) nodes, and atrioventricular conduction^46^**.** Our findings showing the decrease in the HR along with the progressive increase in R-wave amplitude and P-R interval elongation were consistent with the results of a previous study, which reported that catecholamines-induced cardiotoxicity indicated the toxic effects on atrioventricular (AV) and sinoatrial (SA) nodes, destruction of myocytes, and/ or myocardial infarction^47^**.**

The majority of myocardial alterations can be attributed to increased oxidative stress. Furthermore, the increased production of myocardial oxidants has been suggested as the main cause of heart failure. The malfunction of the Na+/K+ ATPase pump and myocardium-imbalanced electrolyte levels has also been implicated^48^**.** Consistent with these findings, our data revealed that ZnO NP-induced cardiotoxicity is associated with oxidative stress development and impairment in antioxidant balance, which occurred in a dose-dependent manner as the highest dose group (T3) showed a highly significant increase in heart NO and lipid peroxidation (MDA) levels. Furthermore, there was a significant depletion in heart GSH levels. Meanwhile, treatment with ZnO bulk did not show any significant changes in either heart NO or lipid peroxidation levels in the heart tissues of rats. Additionally, no significant change in the GSH levels was seen, confirming its safety. These results also supported our previous findings on the hepatotoxic effect of ZnO NPs in rats where we showed increased oxidative stress production and depletion in antioxidant levels in both serum and tissues^14^.

ROS plays a crucial role in the impairment of the cardiomyocyte microenvironment due to exposure to NPs, which was proved by increased ROS production, altered redox homeostasis, and diminished antioxidant ability, resulting in damage to the cardiac muscles^8^**.** The overproduction of NO levels in tissues can lead to severe cellular damage. NO may cause constriction of coronary arteries and contribute to myocardial ischemia in patients with coronary artery disease, also NO could be relevant to oxidative stress and or nitrosative stress processes due to its chemical nature when it is excessively produced it could participate in the formation of biologically reactive nitrogen oxide species (RNOS) that are mediated in oxidative nitration and oxidative damage of biomolecules such as lipids, proteins, and DNA^13^.

Zinc is an essential nutrient for human health and is vital for the physiological functions of various organs, owing to its important role in enzymes and proteins^49^. ZnO NPs were reported to induce more toxic effects than larger (bulk) ZnO particles due to their larger surface-to-mass ratio, which enhanced their solubility in biological systems^50^. Heavy metals enhance toxicity when they cannot be metabolized in the body and are accumulated in soft tissues^51^. Our findings showing the estimated zinc concentrations in heart tissues shed light on the mechanism of ZnO NPs cardiotoxicity.

The results showed that injection with the highest dose of ZnO NPs induced a highly significant increase in zinc ion (Zn++) concentrations in the heart tissues, whereas the bulk-treated group showed a nonsignificant increase in heart Zn++ concentrations compared to the control. As discussed previously, the accumulation of Zn++ in the hepatic tissues due to the administration of the same dose of ZnO NPs was associated with other biochemical and histological indices of the liver of rats^14^**.**

Recent studies revealed that increased production of Zn2+ during the excitation–contraction cycle in the cardiomyocytes indicated oxidative stress, which could trigger further production of pro-oxidants leading to oxidative damage in cardiomyocytes and heart dysfunction^52^**.**

The histopathological assessment of the rats' heart sections showed dose-dependent alterations in the cardiac tissues of rats as the cardiac sections of the T3 group (highest dose) exhibited highly marked vascular congestion and degeneration along with myofibre dislocation, confirming the biochemical and electrophysiological effects of the ZnO NPs. While the T1 and T2 groups showed mild degeneration and congestion, the bulk group exhibited normal cardiomyocytes, supporting our biochemical findings. Consistent with our results, a report showed that oral administration of 15 nm-sized ZnO NPs in rats caused significant pathological effects in the heart, including degeneration of the heart muscles, focal hemorrhages, and bleeding along with severe anemia^53^**.**

## Conclusion

In conclusion, this study demonstrated that the subacute i.p. injection of ZnO NPs induced cardiac dysfunctions and pathological lesions in a dose-dependent manner, whereas the ZnO bulk form did not exert any toxicity. Our data also suggest that the accumulation of Zn++ in the heart tissues, accompanied by increased oxidative stress and depletion in the antioxidant activity can be the possible underlying mechanisms behind ZnO NPs’ toxic effects on both the mechanical and electrical activities of the cardiomyocytes. However, further in-depth investigation is needed at the molecular level to fully understand the mechanism of ZnO NPs’ cardiotoxicity.

## Data Availability

All data generated or analysed during this study are included in this published article. Further information and requests for resources and supporting data will be fulfilled by the corresponding authors. The ZnO NPs (nanorods) powder was prepared in the Department of Physics, Faculty of Science, Suez University, Suez, Egypt, using microwave irradiation. The reagents and the ZnO bulk material (particle size = 5 μm) were purchased from Sigma-Aldrich, Germany. The ZnO NPs and bulk material suspensions were freshly prepared using physiological saline (0.9% sodium chloride). The details about the preparation and characterization of the ZnO nanomaterials and bulk suspensions are available in our previous study^14^.

## Abbreviations

ZnO NPs: Zinc oxide nanoparticles
OS: Oxidative stress
ROS: Reactive oxygen species
LDH: Lactate dehydrogenase
CK-MB: Creatine kinase-MB
ECG: Electrocardiography
HR: Heart rate
AV node: Atrioventricular node
GSH: Reduced glutathione
MDA: Malondialdehyde
NO: Nitric oxide
Zn++: Zinc ions
H&E: Hematoxylin and eosin
SEM: Scanning electron microscope
TEM: Transmission electron microscope
IP: Intraperitoneal
